# Jigsaw Puzzles As Cognitive Enrichment (PACE) - the effect of solving jigsaw puzzles on global visuospatial cognition in adults 50 years of age and older: study protocol for a randomized controlled trial

**DOI:** 10.1186/s13063-017-2151-9

**Published:** 2017-09-06

**Authors:** Patrick Fissler, Olivia C. Küster, Laura S. Loy, Daria Laptinskaya, Martin J. Rosenfelder, Christine A. F. von Arnim, Iris-Tatjana Kolassa

**Affiliations:** 1 Institute of Psychology and Pedagogy, Clinical and Biological Psychology, Ulm University, Albert-Einstein-Allee 47, D-89081 Ulm, Germany; 2 Department of Neurology, Ulm University, Oberer Eselsberg 45, D-89081 Ulm, Germany; 3 School of Communication, Media Psychology (540 F), University of Hohenheim, D-70593 Stuttgart, Germany

**Keywords:** Jigsaw puzzles, Visuospatial cognition, Cognitive aging, Cognitive intervention, Cognitive enrichment, Dementia, Cognitive impairment, Daily functioning

## Abstract

**Background:**

Neurocognitive disorders are an important societal challenge and the need for early prevention is increasingly recognized. Meta-analyses show beneficial effects of cognitive activities on cognition. However, high financial costs, low intrinsic motivation, logistic challenges of group-based activities, or the need to operate digital devices prevent their widespread application in clinical practice. Solving jigsaw puzzles is a cognitive activity without these hindering characteristics, but cognitive effects have not been investigated yet. With this study, we aim to evaluate the effect of solving jigsaw puzzles on visuospatial cognition, daily functioning, and psychological outcomes.

**Methods:**

The pre-posttest, assessor-blinded study will include 100 cognitively healthy adults 50 years of age or older, who will be randomly assigned to a jigsaw puzzle group or a cognitive health counseling group. Within the 5-week intervention period, participants in the jigsaw puzzle group will engage in 30 days of solving jigsaw puzzles for at least 1 h per day and additionally receive cognitive health counseling. The cognitive health counseling group will receive the same counseling intervention but no jigsaw puzzles. The primary outcome, global visuospatial cognition, will depict the average of the *z*-standardized performance scores in visuospatial tests of perception, constructional praxis, mental rotation, processing speed, flexibility, working memory, reasoning, and episodic memory. As secondary outcomes, we will assess the eight cognitive abilities, objective and subjective visuospatial daily functioning, psychological well-being, general self-efficacy, and perceived stress. The primary data analysis will be based on mixed-effects models in an intention-to-treat approach.

**Discussion:**

Solving jigsaw puzzles is a low-cost, intrinsically motivating, cognitive leisure activity, which can be executed alone or with others and without the need to operate a digital device. In the case of positive results, these characteristics allow an easy implementation of solving jigsaw puzzles in clinical practice as a way to improve visuospatial functioning. Whether cognitive impairment and loss of independence in everyday functioning might be prevented or delayed in the long run has to be examined in future studies.

**Trial registration:**

ClinicalTrials.gov, NCT02667314. Registered on 27 January 2016.

**Electronic supplementary material:**

The online version of this article (doi:10.1186/s13063-017-2151-9) contains supplementary material, which is available to authorized users.

## Background

Neurocognitive disorders such as mild cognitive impairment and dementia become increasingly important healthcare issues [1]. Engagement in cognitively challenging activities is associated with a reduced risk for future cognitive impairment [2] and dementia [3]. It is among the factors with the highest projected impact on the prevalence of Alzheimer’s dementia [4]. Randomized controlled trials show beneficial cognitive effects of diverse cognitive activities such as cognitive training [5–9], video games [10–13], card and board games [14, 15], and educational programs such as computer, digital photography, or theater courses [16–18]. However, these evidence-based cognitive activities are characterized by obstacles such as high financial costs, low intrinsic motivation, logistic challenges of group-based activities, or the need to use digital devices. We believe that these characteristics of currently investigated cognitive activities reduce their feasibility and implementation in clinical practice.

Solving jigsaw puzzles is a cognitively challenging activity, especially within the visuospatial cognitive domain. We assume that visuospatial cognitive demands comprise perception, constructional praxis, mental rotation, processing speed, flexibility, working memory, reasoning, and episodic memory. In contrast to other cognitive activities, mentioned above, it depicts a low-cost, intrinsically motivating leisure activity which can be executed alone or with others and without the need to operate digital devices. However, to our knowledge, the effect of solving jigsaw puzzles on cognition has not been investigated yet. Next to the positive impact of a wide range of cognitively challenging activities on cognition, several other findings indicate the potential of jigsaw puzzles to promote visuospatial cognitive health. Performance in solving jigsaw puzzles is highly correlated with performance in visuospatial reasoning tasks [19]; jigsaw puzzle play in preschoolers is associated with future visuospatial transformation skills [20]; and engagement in intellectual activities including jigsaw puzzles predicts a reduced risk of Alzheimer’s dementia [21–23]. Thus, the primary aim of this study is to evaluate the effect of solving jigsaw puzzles on visuospatial cognition.

Furthermore, the effects of cognitive interventions on daily functioning and psychological outcomes are scarcely investigated [24–26]. As secondary aims, this study thus examines the effects of solving jigsaw puzzles on visuospatial everyday functioning such as instrumental activities of daily living and self-reported cognitive failures, and on psychological outcomes such as well-being, general self-efficacy, and perceived stress.

## Methods

The Jigsaw *P*uzzles *A*s *C*ognitive *E*nrichment (PACE) study will take place at Ulm University, Germany, as a randomized, active-controlled, assessor-blinded superiority trial with two parallel groups. Between March and approximately October 2016, we will include and randomly assign 100 cognitively healthy, middle-aged and older adults (age 50 years and above) to a jigsaw puzzle group or a cognitive health counseling group with a 1:1 allocation ratio in blocks of four, stratified by cognitive status and age. Participants in the jigsaw puzzle group will engage in 30 days of solving jigsaw puzzles (6 days/week over a period of 5 weeks for at least 1 h/day) and receive cognitive health counseling (15-minutes face-to-face counseling plus three telephone calls). The cognitive health counseling group will receive the same counseling intervention but will not solve any jigsaw puzzles during the intervention period. The SPIRIT 2013 Checklist provides an overview about the contents of this study protocol (see Additional file 1).

### Procedure

**Fig. 1:**
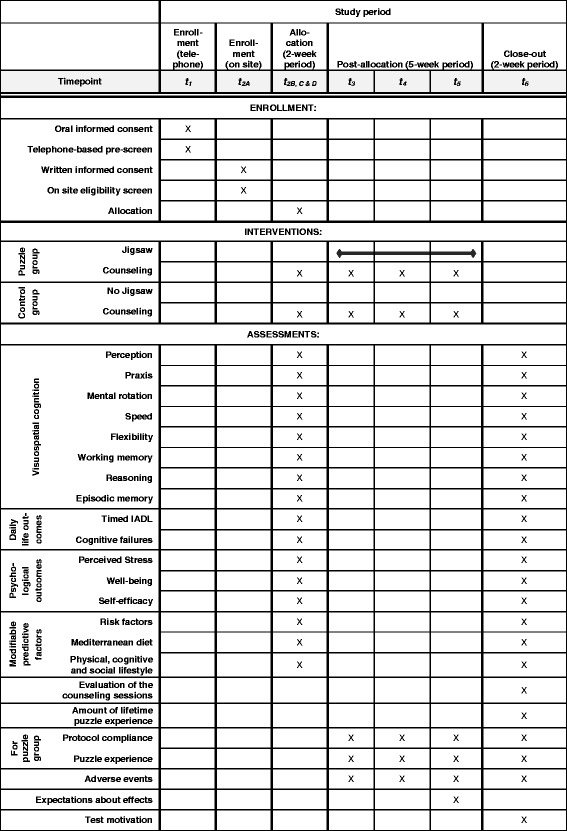
SPIRIT schedule of enrollment, interventions, and assessments. IADL instrumental activities of daily living

**Fig. 2:**
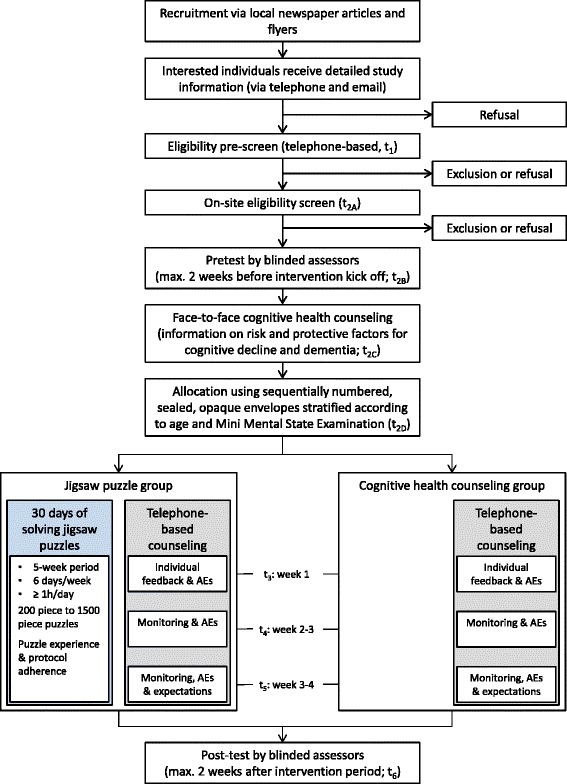
Flow chart of the study procedures. Expectations are the participants’ expectations about benefits in visuospatial cognitive performance. AE adverse event

The Ethics Committee of Ulm University approved this study. We will invite individuals interested in participating in the study to a telephone-based interview. Here, they will receive detailed study information and give oral informed consent. A pre-screening will assess eligibility (t_1_, see Figs. 1 and 2). At an appointment at Ulm University, successfully pre-screened participants will be able to give written informed consent and will undergo further screening (t_2A_). For included participants, the procedure will consist of a 1.5-h pretest assessment composed of neuropsychological tests and questionnaires (t_2B_) followed by the face-to-face cognitive health counseling (t_2C_). Subsequently, non-blinded study staff will disclose the group allocation to the participants (t_2D_). Within 2 weeks after the baseline assessment, the 5-week intervention period will start. We will contact all participants three times via telephone for cognitive health counseling and the assessment of adverse events (AEs, t_3_ – t_5_). These telephone-based interviews will also serve to monitor the jigsaw puzzle experience and protocol adherence in the jigsaw puzzle group. Within 2 weeks after the intervention period, we will assess the same outcomes as in the pretest assessments in the 1.5-h posttest assessment at Ulm University (t_6_). Psychologists who are blind to group allocation will conduct the pretest and posttest assessments.

### Participants

The study aims to include 100 participants who we will recruit via local newspaper articles and flyers. Inclusion criteria are 50 years of age or older, unimpaired cognition defined by a Mini-Mental State Examination (MMSE) [27] score ≥ 24, the commitment to invest at least 1 h per day for 30 days in solving jigsaw puzzles within a 5-week period, an interest in solving jigsaw puzzles, and low jigsaw puzzle experience in the past 5 years (fewer than five completed jigsaw puzzles).

Exclusion criteria are the participation in another interventional study and self-reported, uncorrected impairment in vision (e.g., red-green color blindness) or motor function of the upper extremity (e.g., hand tremor) that considerably impairs jigsaw puzzle performance. In addition, individuals with any self-reported psychiatric, neurological, or other diseases that could affect cognitive change over time will be excluded. These diseases comprise current major depression, dementia or Parkinson’s disease, a history of stroke with current impairment, epilepsy, multiple sclerosis, or a current malignant tumor. Apart from the cognitive impairment, which we will assess at the on-site screening (t_2A_), telephone-based pre-screening will examine inclusion and exclusion criteria after oral informed consent (t_1_, see Figs. 1 and 2).

### Measures

All outcomes of the study are continuously scaled and we will assess the mean change between baseline (t_1_) and posttest assessments (t_6,_ approximately 5 weeks after t_1_). The outcome domains and their specific measurement, and the assessment of participants’ expectations, predictive variables of cognitive decline, and adverse events are described in the following sections.

#### Primary outcome

A composite score of global visuospatial cognition will depict the primary outcome reducing α-error inflation [28]. This score will constitute the average of the eight, *z*-standardized visuospatial cognitive ability scores that include visual perception, visuoconstruction, mental rotation, processing speed, flexibility, working memory, reasoning, and episodic memory (see “Secondary outcomes”). We will calculate the individual composite scores, if at least five cognitive ability scores are available for both pretest and posttest for an individual. Analysis will be based on individuals for whom both pretest and posttest ability scores are available. We will *z*-standardize all test scores based on pretest means and standard deviations.

#### Secondary outcomes

Secondary outcomes of visuospatial cognition will include the eight visuospatial cognitive ability scores. An adapted version of Benton’s Judgment of Line Orientation Test will assess visual perception [29]. In addition to Benton’s 30 original test items, the version in this study will also include five new test items of greater difficulty to differentiate performance in the higher performance range (visual perception score: number of correct items). The Rey Complex Figure Test (pretest) [30] and the Taylor Complex Figure Test (posttest) [31] will serve to evaluate visuoconstruction and visuospatial episodic memory. Pretest and posttest assessments will apply different figures to minimize the risk that recall of the pretest figure affects posttest performance (visuoconstruction score: points in the copy trial; episodic memory score: sum of the points in the immediate and delayed free recall trials; two raters (DL and HA) will rate all trials; points for each trial depict the mean of both ratings). The Mental Rotations Test (MRT)-Letters (12 items; maximum (max.) time: 90 s) adapted from [32] and the MRT-A (items 1–12; max. time: 240 s) [33] will measure 2D and 3D mental rotation performance, respectively, in order to produce a score of mental rotation ability (2D and 3D sub-scores: seconds per correct item; in the case of no correct item, the score will depict twice the max. time; mental rotation score: average of both *z*-standardized sub-scores). The Trail Making Test (TMT) A and B will assess visuospatial processing speed and flexibility, respectively (both ability scores: seconds per correct connection; max. time: 180 s for TMT A and 300 s for TMT B) [34, 35]. The Visual Memory Span from the German edition of the Wechsler Memory Scale-Revised [36] will serve to measure visuospatial working memory (working memory score: sum of points for forward and backward block tapping). Finally, we apply the Block Design subtest from the German edition of the Wechsler Adult Intelligence Scale-III to assess visuospatial reasoning (reasoning score: sum of points) [37].

The study assesses secondary visuospatial daily functioning outcomes both objectively and subjectively. The objective assessment of daily life functioning comprises three visuospatial subtests of the Timed Instrumental Activities of Daily Living (TIADL) [38, 39] (sub-scores: seconds for correct completion of task A - finding a telephone number*,* task B - making change*,* and task C - reading the list of ingredients on cans; objective daily functioning score: average of all three *z*-standardized sub-scores). Eleven visuospatial items of the Cognitive Failures Questionnaire will assess subjective function in everyday life (subjective daily functioning score: sum of points) [40, 41].

Self-report questionnaires will serve to assess secondary psychological outcomes. We will measure psychological well-being during the past 2 weeks with the World Health Organization (WHO)-5 Well-Being Index [42], general self-efficacy with the Generalized Self-Efficacy Scale [43], and perceived stress during the past month with the Perceived Stress Scale-14 [44].

We will use a 40-piece jigsaw puzzle (image: Rothenburg ob der Tauber by Ravensburger Spieleverlag GmbH (RSV); piece size: ca. 2.45 × 2.4 cm) to measure jigsaw puzzle skill. Thereby participants will complete the frame first and then the inner section of the puzzle (sub-scores for frame and inner section: seconds per connected piece; max. time for each task: 300 s; jigsaw puzzle score: average of both sub-scores).

In case of floor or ceiling effects (skewness ≥ 1 or ≤ − 1), we will use skewness minimization transformations (Blom transformations [45]) for all relevant ability scores and sub-scores. To increase measurement reliability and therefore the power of the study, the same psychologist will conduct pretest and posttest assessment of each participant, if possible. In addition, we will apply different methods to detect data entry errors, including range checks, plotting of pretest and posttest scores, and assessment of the retest-reliability for each score. Low retest-reliability of the primary outcome (*r* < .80) will result in double data entry.

#### Participants’ expectations

To account for potential effects of participants’ expectations regarding cognitive benefits of the interventions, we will assess these expectations within the last telephone call between week 3 and 4 after the start of the intervention (t_5_, see Figs. 1 and 2). Participants will be asked (1) whether they expect that their performance in the visuospatial cognitive tests has been influenced positively since the pretest (answers: “yes” or “no”), and (2) how much they expect their performance in the visuospatial cognitive tests to change from pretest to posttest assessment on a 5-point rating scale (answers range from “improve markedly” to “decline markedly”). We will use the same questions with respect to participants’ expectations of improvement in jigsaw puzzle performance.

#### Predictive variables of cognitive decline and dementia

The investigators will obtain the predictive socio-demographic variables age, gender, years of education, and main profession, using interview-based questionnaires. Three questions on the subjective degree of jigsaw puzzle experience on a 5-point rating scale (answers range from “very high” to “not at all”), the number of jigsaw puzzle hours, and the number of connected jigsaw puzzle pieces within participants’ lifetime will assess lifetime jigsaw puzzle experience. The assessment of risk factors for cognitive decline and dementia with self-rating questionnaires will include items on high blood pressure, hypercholesterolemia, diabetes mellitus, smoking, alcohol misuse, and obesity. The newly developed Challenging Leisure Activity Questionnaire is based on the CHAMPS Physical Activity Questionnaire for Older Adults [46] and will serve as a measure of the protective factors social, physical, and cognitive activities. Participants will indicate for each of 44 socially, physically, and cognitively challenging activities whether they performed them at least once during the past 4 weeks. A short 14-item Mediterranean diet questionnaire [47] will assess components of a Mediterranean diet. The measurement of risk and protective factors will serve as the basis for individual feedback in the cognitive health counseling and for the assessment of behavior changes between pretest and posttest assessments.

#### Adverse events

Non-blinded study staff will address potential adverse events at the beginning of the three telephone-based cognitive health counseling sessions (t_3_, t_4_, t_5_; see Figs. 1 and 2) and after completion of the posttest outcome measures.

### Interventions

Participants will be randomly assigned to one of two groups, the jigsaw puzzle group or the cognitive health counseling group (see Fig. 2). The jigsaw puzzle group will receive the 30-day jigsaw puzzle intervention and the cognitive health counseling, while the cognitive health counseling group will receive the cognitive health counseling only. We will ask the participants of the cognitive health counseling group not to solve any jigsaw puzzles within the 5-week intervention period. Participants of the cognitive health counseling group will receive a jigsaw puzzle after completion of the posttest assessments.

#### Cognitive health counseling

All participants will receive a 15-minute session of cognitive health counseling in an individual face-to-face setting, including a take-home brochure (see Figs. 1 and 2). The counseling will inform about modifiable protective factors and risk factors for cognitive decline and dementia according to current research evidence. It will be based on the guidelines of the German Association for Psychiatry, Psychotherapy, and Psychosomatics on dementia. In particular, the counselors will explain protective effects of physical, cognitive, and social activities, and a Mediterranean diet. The counseling will further inform about the modifiable risk factors obesity, hypertension, diabetes mellitus, high cholesterol and homocysteine levels, smoking, and alcohol misuse.

In addition, the cognitive health counseling will comprise three telephone calls during the 5-week intervention period. In the first call (t_3_; week 1), participants will receive individual feedback based on their responses in the questionnaires on modifiable risk and protective factors at the pretest assessment. Here, we will give information about potential behavior changes and ask the participants whether they are motivated to adapt any of them. In the second and third telephone-based interview, we will ask for changes in behavior with respect to the predictive factors. Taken together, the behavior change techniques of the cognitive health counseling include the shaping of knowledge about cognitively healthy behaviors (t_2_), providing individual feedback on these behaviors (t_3_), and monitoring behaviors by the study staff with participants’ awareness (t_4_ and t_5_, cf. [48]).

#### Jigsaw puzzle intervention

Participants of the jigsaw puzzle group will solve jigsaw puzzles on 6 days a week for 5 weeks for at least 1 h per day. Every participant will get the same 300 piece jigsaw puzzle at first (*Beautiful Prague* by RSV; piece size: 2.45 × 2.4 cm). The participants will select subsequent puzzles out of a catalogue with 200, 300, 500, 1000, and 1500 piece jigsaw puzzles of the RSV adult puzzle 2016 collection (piece sizes included: 2.8 × 3.0 cm; 2.45 × 2.4 cm; 2.0 × 1.8 cm; 1.9 × 1.7 cm; and 1.8 × 1.7 cm, respectively).

The intervention will take place at participants’ homes. They will protocol their jigsaw puzzle time in a diary immediately after each jigsaw puzzle session. On each jigsaw puzzle day, participants will note the jigsaw puzzle name, the jigsaw puzzle duration, and if necessary additional comments about their experiences. Immediately after each of the three telephone-based cognitive health counseling sessions, participants will report the content of the diary for the monitoring of the protocol adherence and of the jigsaw puzzle performance (time per connected piece). Finally, the telephone-based interview includes questions about participants’ jigsaw puzzle experience for each solved jigsaw. Jigsaw puzzle experience will include the desire to solve the jigsaw puzzle, the pleasure in solving the jigsaw puzzle, the experienced competence of solving the jigsaw puzzle, and the perceived difficulty of the jigsaw puzzle. We will evaluate all four measures with a single question and a 5-point rating scale that ranges from “very high” to “not at all”. Participants will be able to freely choose their preferred jigsaw puzzles. However, we will recommend them to constantly increase the difficulty and challenge of the jigsaw puzzles through the increase of pieces per puzzle - which is accompanied with a decrease in piece size - as long as this does not reduce their pleasure and fun. These recommendations will depend on the reported perceived difficulty, challenge, pleasure, and fun, and their jigsaw puzzle performance (time per connected piece). If participants report adverse conditions associated with solving jigsaw puzzles (e.g., muscle pain during jigsaw puzzling or the development of a strong craving to solve jigsaw puzzles), the study staff will recommend to reduce puzzle time, not to stick to the protocol of at least 1 h per day, or to stop solving jigsaw puzzles as appropriate.

### Randomization

We will use stratified, blocked randomization to avoid baseline differences in age and cognitive status between the cognitive health counseling group and the jigsaw puzzle group, while at the same time achieving groups of similar sizes. We will stratify participants into two age bands (50–64 years and 65 years and older) and two cognitive status bands (MMSE: 24–27 and 28–30) resulting in four strata (stratum 1, age ≥ 64 years, MMSE 28–30; stratum 2, age ≥ 64 years, MMSE 24–27; stratum 3, age 50–64 years, MMSE 28–30; and stratum 4, age 50–64 years, MMSE 24–27). In each stratum, randomized allocation of the participants will happen in blocks of four (two participants to each group) using the online randomization software random.org (https://www.random.org/). LSL will generate the random allocation sequence for each stratum and conceal it in sequentially numbered opaque envelopes which we will store in a separate box for each stratum. OCK, PF, and DL will conduct the telephone-based pre-screening. DL, MJR, or HA will enroll participants after on-site assessment of the MMSE inclusion criterion and conduct the blinded assessment. OCK or PF will conduct the face-to-face cognitive health counseling. Immediately afterwards, they will disclose group assignment. Thus, the research staff responsible for enrollment and the first part of the cognitive health counseling will be blinded to group assignment and not able to predict future group assignment.

### Blinding and strategies to deal with expectation and motivation effects

Outcome assessors will be blinded to group allocation of participants. To prevent disclosure of group assignment by participants, we will remind them at the last telephone interview (t_5_) not to bring the solved jigsaw puzzles to the posttest assessment. Second, we will remind them at the last telephone interview (t_5_) and immediately before the posttest assessment (t_6_) not to tell the assessor anything that may give hints to the allocated group. Here, the participants will also sign a confirmation statement that they received this information. Blinding participants to the assigned group is by nature not feasible. Given the lack of a double-blind design, several strategies will serve to reduce potentially different expectations and motivation between groups [49]. First, implementing an additional component in both groups (i.e., cognitive health counseling) aims to induce similar positive expectations regarding cognitive benefits (cf. component control design; [49, 50]). Second, the investigators will tell all participants before disclosure of group assignment that (a) both groups should benefit cognitively from the cognitive health counseling and that (b) it is currently not known whether solving jigsaw puzzles has a positive impact on cognition. Third, participants of both groups will receive the same amount of study staff contact during the intervention period (apart from questions regarding jigsaw puzzle experiences and adherence) and the same opportunities to express their concerns and to receive attention (face-to-face health counseling, three telephone calls, and the possibility to contact the study staff whenever wished). As differential expectations between groups are still likely to some degree and cannot be completely excluded, the study assesses expectations on a continuous and on a categorical scale in the last telephone call approximately 9 days before the follow-up test (see confounding variables section). We avoid an assessment at the follow-up, as this might either influence test performance through activated expectations (when assessed directly before the neuropsychological tests) or might alter expectations according to test performance (when assessed directly after the neuropsychological tests) [49]. In addition, we will aim to keep test motivation high in both groups by asking participants again, directly before the follow up, whether they want to receive their normed individual test results after study completion. One item on a 5-point rating scale at the posttest assessment (t_6_) will assess test motivation.

### Prevention of dropouts, noncompliance, and missing data

To ensure the application of the intention-to-treat principle, great effort must be invested to prevent dropouts, noncompliance, and missing outcome data, which introduce deviation from the randomization scheme [51, 52]. We will use different strategies including good personal contact through the telephone-based counseling; payment of 20€ for pretest and posttest assessments; communication of personal neuropsychological test results after the posttest assessments; interest in solving jigsaw puzzles and time commitment as study inclusion criteria; good availability of appointments for the pretest and posttest assessments (if appointments are not possible within the 2-week pretest and posttest period, we will arrange them as close as possible to this period); reminders for the posttest appointment at the last telephone-based counseling (t_5_); also if participants do not adhere to the intervention protocol, we will ask them to complete the posttest assessment; construction of a global ability score that allows some amount of missing data in the neuropsychological test battery; and prevention of excessive and insufficient mental demands through a large number of jigsaw puzzle difficulty levels (200 to 1500 piece puzzles; 2.8 × 3.0 cm to 1.8 × 1.7 cm piece sizes), adapted according to participants’ preferences, jigsaw puzzle experiences, and performance.

### Statistical analysis

The primary efficacy analysis will be based on mixed-effects models' group × time interaction [53] in an intention-to-treat approach [54] that includes all randomly assigned participants with follow-up observations. Effect sizes will include standardized differences in the pretest-posttest change scores between both groups. Standardization will be based on (1) baseline scores and (2) pretest-posttest change scores. In additional analyses, we will account for potential confounding variables such as baseline performance, the number of challenging social, physical, and cognitive activities performed in the past 4 weeks, the pretest-posttest change in these activities, Mediterranean diet in the past 4 weeks, and pre-posttest change in diet, age, education, participants’ test motivation at the posttest assessment, and their expectations of cognitive benefits.

As the primary intention-to-treat analysis is a conservative method, which may underestimate the true effect size of solving jigsaw puzzles for individuals who follow the protocol, we will use a supportive per-protocol analysis, which includes only participants who completed at least 80% of the jigsaw puzzle protocol (≥24 jigsaw puzzle days with a minimum of 45 min). With this supportive analysis, we do not only assess the robustness of effects, but may also receive a more accurate estimate of the true effect size given optimal conditions.

For subgroup and moderator analyses, pre-specified variables comprise baseline global visuospatial cognition, amount of lifetime puzzle experience, number of challenging social, physical, and cognitive activities within the intervention period, age, MMSE, and experienced pleasure in solving jigsaw puzzles.

Finally, we will assess cognitive demands of solving jigsaw puzzles and the transfer potential of gains in jigsaw puzzle skill on gains in the study outcomes by calculating the correlation between jigsaw puzzle skill in the 40-piece puzzle and the study outcomes at baseline.

#### Power analysis

The probability to find a significant effect with 50 included participants per group, a dropout rate of 15%, a small true effect size of *f* = 0.1, pretest-posttest correlation of *r* = .85, and an α-error of 0.05 is 90%. We used G*Power 3.1.6. for power analysis [55].

## Discussion

Our upcoming findings might have a clinical implication for adults over 50 years of age who have low puzzle experience in the past 5 years but are interested in solving jigsaw puzzles. As solving jigsaw puzzles depicts a low-cost, intrinsically motivating leisure activity, which can be executed alone or with others, and without the need to operate a digital device, positive results would indicate a highly feasible cognitive intervention to improve visuospatial cognition and everyday functioning, and psychological outcomes in this population. We will not investigate the effect of jigsaw puzzling on overall cognitive functioning including cognitive domains such as language or verbal working memory as generalization to non-trained cognitive domains cannot be expected from a theoretical and an empirical point of view [56, 57]. However, as visuospatial dysfunctions (e.g., deficits in visuoconstruction or visuospatial episodic memory and missed traffic signs or relocated objects) are common in neurocognitive diseases such as mild cognitive impairment and dementia - especially due to Alzheimer’s disease, dementia with Lewy bodies, and posterior cortical atrophy [23] - lifelong jigsaw puzzle experience might be one out of many cognitive activities that may contribute to a delayed clinical manifestation of neurocognitive disorders.

Limitations of the study design are the lack of a long-term follow-up and of the investigation of biological mediators, and the lack of participants’ blinding to group allocation, which could lead to differential expectation effects. In addition, the selected outcomes may have limitations in assessing the effects of jigsaw puzzling on cognitive abilities and daily functioning. Finally, the cognitive health counseling might have different behavioral effects in the counseling and the puzzle group that could mask the effects of jigsaw puzzling.

To address the lack of long-term follow-up, we will ask participants after study completion whether they will be interested in future long-term follow-up that we aim to conduct approximately 1.5 years after posttest assessment.

Blinding of participants is by nature not feasible in behavioral intervention studies as participants are always aware of what they are doing. Thus, expectation effects can never be fully excluded. Multiple approaches to minimize expectation effects will be used and participants’ expectations with regard to performance improvement will be measured in a telephone-based interview approximately 9 days prior to the posttest assessment. Thus, it will be possible to statistically account for potential expectation effects.

Even if not expected, the selected outcomes of the study may not cover the cognitive abilities that are actually engaged and improved through jigsaw puzzling (e.g., verbal working memory) or may include abilities that are not recruited and improved through the intervention (e.g., cognitive flexibility). To prevent this problem, the selection of the outcomes was based on a cognitive task analysis of jigsaw puzzling that indicated the expected cognitive demands [58, 59]. Furthermore, we will assess the association between the outcome measures and jigsaw puzzle skill at baseline, in order to validate the recruited cognitive abilities of jigsaw puzzling [60] and the transfer potential of gains in jigsaw puzzle performance on gains in the respective outcomes [61]. Finally, there is a not fully preventable risk that gains in outcomes may be a result of skill acquisition or newly learned strategies rather than the improvement of broad cognitive abilities [14]. However, in contrast to many previous cognitive training studies, we use a large set of transfer tasks and these task paradigms are substantially dissimilar from the training task. Thus, we avoid the risk that skill-induced or strategy-induced gains in single and structurally similar tasks with the training task will be misinterpreted as gains in broad cognitive abilities.

Finally, we cannot fully exclude that the cognitive health counseling intervention that is conducted in both groups may have a differential effect between the groups. However, we will assess this potential by evaluating group differences in behavioral changes in cognitive, physical, and social activities, and in Mediterranean diet. In additional analyses of the group effect on the study outcomes, we will statistically account for these behavioral changes (see “Statistical analysis”).

In the case of positive results, future research should investigate maintenance of effects, biological mechanisms of effects, potential effects in other populations such as people with impairments in visuospatial cognition, and effects of long-term experience in solving jigsaw puzzles on the prevention of neurocognitive disorders such as mild cognitive impairment and dementia.

## Additional file

Additional file 1: SPIRIT 2013 Checklist: Recommended items to address in a clinical trial protocol and related documents. (DOC 126 kb)

## Abbreviations

AE: Adverse event
MMSE: Mini-Mental State Examination
MRT: Mental Rotations Test
PACE: Jigsaw Puzzles As Cognitive Enrichment
RSV: Ravensburger Spieleverlag GmbH
TIADL: Timed Instrumental Activities of Daily Living
TMT: Trail Making Test
